# A predictive model for early intubation in patients with COVID–19–induced acute hypoxemic respiratory failure under awake prone position

**DOI:** 10.1186/s13613-025-01602-4

**Published:** 2025-11-24

**Authors:** Luis Morales–Quinteros, Nora Angélica Fuentes, Alfonso Muriel, Matías Olmos, Marina Busico, Alejandra Vitali, Adrián Gallardo, Erika P. Plata–Menchaca, Ricard Ferrer, Antonio Artigas, Mariano Esperatti

**Affiliations:** 1 Intensive Care Department, Vall d’Hebron University Hospital, Vall d’Hebron Institut de Recerca, Passeig de la Vall d’Hebron, 119–129, 08035 Barcelona, Spain; 2 CIBER of Respiratory Diseases [CIBERES], Institute of Health Carlos III, Madrid, Spain; 3 Intensive Care Unit, Hospital Privado de Comunidad, Universidad Nacional de Mar del Plata, Mar del Plata, Buenos Aires Argentina; 4 Clinical Biostatistics Unit, Hospital Universitario Ramón y Cajal, IRYCIS, CIBERESP, Universidad de Alcalá, Madrid, Spain; 5 Intensive Care Unit, Clínica Olivos SMG, Olivos, Buenos Aires Argentina; 6 Intensive Care Unit, Sanatorio de la Trinidad Palermo, Ciudad Autónoma de Buenos Aires, Argentina; 7 Intensive Care Unit, Sanatorio Clínica Modelo de Morón, Universidad de Morón, Morón, Buenos Aires Argentina; 8 Clinic Barcelona University Hospital Shock, Organ Dysfunction and Resuscitation, Vall d’Hebron Hospital Campus, Barcelona, Spain; 9 Critical Care Center, Institut d’Investigació i Innovació Parc Taulí I3PT– CERCA, Hospital Universitari Parc Taulí, Universitat Autónoma de Barcelona, Sabadell, Spain

**Keywords:** Prone positioning, Acute respiratory failure, High flow nasal cannula, COVID–19, Acute respiratory distress syndrome

## Abstract

**Background:**

Awake prone positioning (APP) reduces the risk of endotracheal intubation and mortality in COVID–19–related acute respiratory failure (ARF) receiving high–flow nasal oxygen (HFNO). However, a significant proportion of patients undergoing APP are ultimately intubated, and mortality in this subgroup remains high. We aimed to develop a predictive model to be applied within the first 24 h of APP to identify patients at higher risk of progressing to intubation within 72 h of APP initiation.

**Methods:**

We conducted a secondary analysis of a prospective multicenter cohort including adult patients with COVID–19–related ARF admitted to six intensive care units in Argentina between June 2020 and January 2021. Eligible patients received HFNO and APP for at least 6 h per day. Physiological variables were collected at ICU admission (baseline) and 24 h after APP initiation. Two multivariable logistic regression models were developed using baseline and 24–hour variables, respectively. Predictors were selected based on clinical relevance and univariable associations. A final model was constructed by integrating variables retained from both time points.

**Results:**

Of 400 patients included, 136 (34%) required intubation within the first 72 h. Patients who required intubation were older, had lower PaO₂ and PaO₂/FiO₂ ratios, and higher respiratory rates both at baseline and after 24 h. The final predictive model included five variables: age, respiratory rate, PaO₂, FiO₂, and SaO₂/FiO₂ ratio, all measured 24 h after APP initiation. A nomogram was developed based on this model to estimate the individual risk of early intubation.

**Conclusion:**

In patients with COVID–19–related ARF treated with HFNO and APP, a model combining baseline characteristics and early physiological response can help predict the need for intubation within 72 h. This tool may support clinicians in identifying high–risk patients and making timely, individualized decisions about escalation of care.

**Supplementary Information:**

The online version contains supplementary material available at 10.1186/s13613-025-01602-4.

## Introduction

Severe acute respiratory failure caused by COVID–19 is characterized by progressive hypoxemia, which often requires invasive mechanical ventilation (IMV). Among ventilated patients, the prognosis is particularly ominous, with mortality rates exceeding 40% [1]. Prone positioning (PP) is an effective intervention to reduce mortality in patients with severe acute respiratory distress syndrome (ARDS) requiring IMV [2, 3]. For this reason, during the SARS–CoV–2 pandemic, the awake prone position (APP) strategy emerged as an alternative therapy aimed at avoiding intubation and its associated adverse outcomes, in the context of the overwhelming surge of cases and the shortage of IMV resources [4]. Subsequently, this strategy was shown to reduce the risk of intubation and mortality, particularly among patients who required some form of non–invasive ventilatory support and had a sufficient duration of exposure to prone positioning [5, 6]. However, a significant proportion of patients undergoing APP eventually are intubated, and mortality in this subgroup also remains high [6]. There is ongoing debate regarding the most appropriate timing for endotracheal intubation in acute respiratory failure managed with non–invasive respiratory support (i.e., occurring earlier or later in the clinical course). Some studies have reported an association between later intubation and worse clinical outcomes [7, 8], while more recent investigations using advanced methodological approaches have challenged the assumption of a causal link [9]. While the causal relationship between intubation timing and clinical outcomes remains complex and context–dependent, the need for timely, individualized decisions in acute care settings persists [10]. In practice, clinicians must often decide whether to continue advanced non–invasive support (i.e., APP) or proceed to intubation based on dynamic physiological parameters assessed within a narrow time window [11]. In this context, predictive tools may support early risk stratification and guide decision–making in patients undergoing APP [12, 13]. Therefore, we aimed to develop a predictive model based on data collected 24 h after APP initiation to identify patients at risk of requiring tracheal intubation within the first 72 h of hospital admission. Additionally, we describe the evolution of physiological parameters during the first 24 h and the relevant clinical outcomes.

## Methods

### Study design

We performed a secondary analysis of a prospective, multicenter cohort conducted in six intensive care units (ICUs) across Argentina during the first and second waves of the COVID–19 pandemic (June 2020 to January 2021) (NCT05178212) [14–16]. Independent institutional review boards approved the original study protocol at each participating center. The study was conducted in accordance with the International Council for Harmonisation Good Clinical Practice (ICH–GCP) guidelines and adhered to the ethical principles outlined in the 2024 revision of the Declaration of Helsinki [17]. This report complies with the TRIPOD (transparent reporting of a multivariable prediction model for individual prognosis or diagnosis) guidelines [18].

### Population and sample size

All consecutive adult patients (aged ≥ 18 years) admitted to the ICU with COVID–19–related acute hypoxemic respiratory failure treated with high–flow nasal oxygen (HFNO) were assessed for eligibility. Acute hypoxemic respiratory failure was defined by the presence of at least one of the following: (a) SpO₂ <92% despite ≥ 4 L/min of supplemental oxygen, (b) signs of increased work of breathing (e.g., accessory muscle use) with a respiratory rate >30 breaths/min, or (c) a PaO₂/FiO₂ ratio < 200 mmHg. Patients were excluded if they had respiratory failure of non–COVID etiology, contraindications to awake prone positioning (APP) (e.g., Glasgow Coma Scale < 9, hemodynamic instability requiring vasopressors, pregnancy, APP < 6 h, PaCO₂ >45 mmHg with pH < 7.35), prior use of positive–pressure ventilation, or required immediate intubation upon ICU admission. All patients meeting the inclusion criteria and none of the exclusion criteria were included. Although no formal sample size calculation was performed, an observed intubation rate of ~ 30% in the original cohort allowed for inclusion of up to 10 predictor variables in multivariable logistic regression analysis, based on the rule of 10 events per variable [19].

### Procedures and variables

Detailed protocols regarding APP implementation, including high–flow oxygen settings, initiation and discontinuation criteria, and positioning procedures, have been previously published [14–16]. Upon ICU admission, inclusion criteria were confirmed, and standardized data collection was initiated using electronic case report forms. Collected variables were organized according to their analytical purpose as descriptive, predictive, or outcome measures. Descriptive variables included demographic and baseline clinical characteristics such as age, sex, weight, height, and the time from ICU admission to initiation of awake prone positioning (APP). APP timing (early vs. delayed) and duration (in the first 24 h and cumulatively) were also recorded. Physiological variables were collected at two time points: at ICU admission (baseline) and 24 h after APP initiation. These included respiratory rate (breaths/min), arterial oxygen pressure (PaO₂), peripheral oxygen saturation (SpO₂), arterial oxygen saturation (SaO₂), and fraction of inspired oxygen (FiO₂). Derived indices included the PaO₂/FiO₂ ratio, the SaO₂/FiO₂ ratio (SAFI), and the ROX index (SpO₂/FiO₂)/respiratory rate) [20]. Variables collected at both time points were used for descriptive purposes; only those obtained 24 h after APP initiation were included as candidate predictors in the multivariable analysis. Additional predictive variables included the absolute change in PaO₂ (ΔPaO₂) and FiO₂ (ΔFiO₂) between baseline and 24 h.

**Fig. 1:**
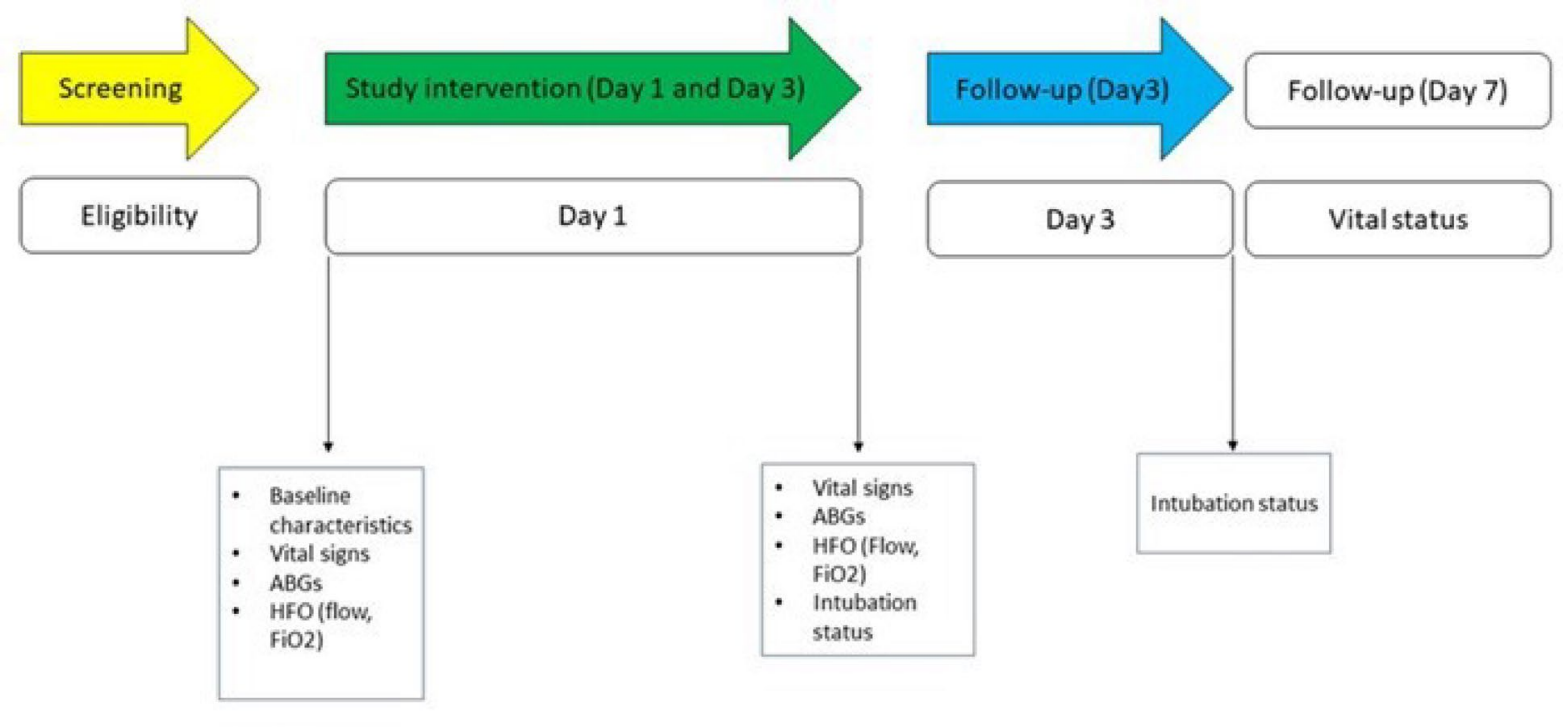
Study overview

Intubation status was recorded within 72 h of ICU admission to assess early treatment failure (Fig. 1).

v

All data were prospectively recorded, anonymized, and stored in a secure database. Data quality was centrally monitored. Variables used in the predictive model were selected a priori based on physiological rationale and clinical relevance.

### Study endpoints

The primary outcome was early intubation, defined as the need for invasive mechanical ventilation (IMV) within 72 h of APP initiation. Additionally, we report other clinically relevant outcomes, including intubation and mortality rates at days 7, and 28 following ICU admission.

The decision to intubate was based on the criteria of the attending healthcare team. However, intubation was recommended for anyone meeting the following criteria: deterioration of neurologic status, hemodynamic instability, or if two or more of the following criteria were met: decline in oxygen saturation with SpO2 < 90% for more than 5 min (not explained by technical failures), lack of improvement in the signs of respiratory muscle fatigue, impossibility to control airway secretions and respiratory acidosis with pH < 7.30 [14, 21].

### Statistical analysis

All statistical analyses were performed by an independent statistician using Stata/MP version 18.0 (StataCorp LLC, College Station, TX, USA). Categorical variables were summarized as frequencies and percentages, and continuous variables as means with standard deviations or medians with interquartile ranges, as appropriate. Group comparisons between intubated and non–intubated patients were performed using the chi–square or Fisher’s exact test for categorical variables, and Student’s *t*–test or Mann–Whitney *U* test for continuous variables.

To identify independent predictors of early intubation (within 72 h), two separate multivariable logistic regression models were constructed: one using baseline variables and another using variables measured 24 h after initiation of awake prone positioning (APP). Candidate predictors were selected based on clinical relevance and univariable associations (*p* < 0.10), and retained through backward elimination. A full model including all candidate variables was also evaluated for robustness. Continuous variables were examined for nonlinearity using multivariable fractional polynomial regression.

Discrimination of each model was assessed using the C–statistic (area under the receiver operating characteristic curve, AUC), and calibration was evaluated using calibration–in–the–large and calibration slope. Internal validation was performed using bootstrap resampling with 1,000 iterations.

A final logistic regression model was constructed by integrating variables retained from both the baseline and 24–hour models. Specifically, five predictors were included: age, respiratory rate, PaO_2_, FiO_2_, and SaO_2_/FIO_2_ (all measured at 24 h). Based on this final model, a nomogram was developed to provide a graphical tool for estimating individual risk of early intubation. Each predictor was assigned a weighted score proportional to its regression coefficient, and total scores were mapped to predicted probabilities using the logistic equation.

To account for different monitoring scenarios, alternative models based on PaO₂/FiO₂, SaO₂/FiO₂, and Δ24h from baseline were developed and their AUCs with 95% CIs were compared with that of the main model to evaluate relative discriminative performance.

A two-sided p value < 0.05 was considered statistically significant. The nomogram was constructed and visualized using Stata.

### Sensitivity analyses

To assess the robustness and potential clinical applicability of our findings, we performed sensitivity analyses through the development of alternative predictive models based on commonly available measures of oxygenation. A PaO₂/FiO₂–based model was developed to represent scenarios in which only arterial blood gases are accessible, while an SaO₂/FiO₂–based model reflected monitoring strategies relying exclusively on oxygen saturation. In addition, a Δ24h-baseline model was generated to capture dynamic changes from baseline values. The discriminative performance of these models was evaluated using the AUC and compared with that of the main model.

## Results

### Study population

During the study period, 1,263 patients with COVID–19 pneumonia and acute respiratory failure (ARF) were admitted to the ICU. Of these, 728 met eligibility criteria for high–flow nasal oxygen (HFNO) and awake prone positioning (APP). After excluding those with less than 6 h per day in APP or with missing data, 400 patients were included in the final analysis.

### Baseline characteristics

**Fig. 2:**
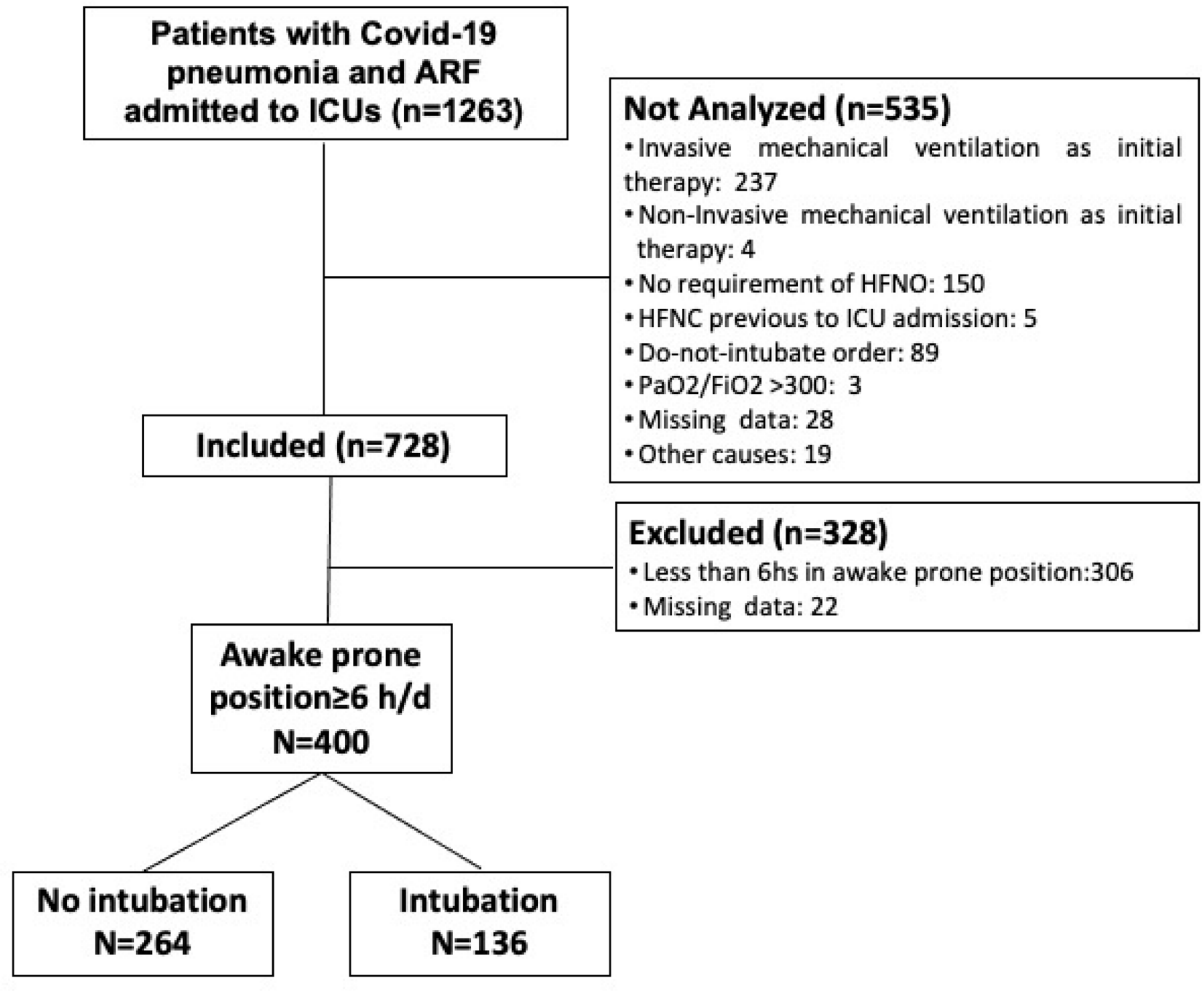
Flowchart of the study participants.* ICU* intensive care unit,* ARF*, acute respiratory failure,* HFNC* high–flow nasal cannula

Among the 400 patients included, 136 (34%) required intubation within the first 3 days, and 264 (66%) remained non–intubated (Fig. 2). Patients who required intubation were significantly older (mean age: 61.5 vs. 53.4 years; *p* < 0.001), had lower baseline PaO₂ (71 vs. 81 mmHg; *p* < 0.001), lower PaO₂/FiO₂ ratio (125 vs. 145; *p* = 0.004), and a lower ROX index (6.6 vs. 7.5; *p* = 0.03). Time from ICU admission to first prone positioning was longer in the intubated group (6 vs. 2 h; *p* < 0.001), and the number of days in prone positioning was shorter (3 vs. 5 days; *p* < 0.001). No significant differences were observed in sex, BMI, flow rate, FiO₂, pH, PaCO₂, SpO₂, SaO₂/FiO₂, heart rate, respiratory rate, or duration of prone positioning on the first day (Table 1).

**Table 1:** Baseline demographic, clinical, and physiological characteristics between intubated and non–intubated patients

Variable	No intubation [*N* = 264]	Intubation [*N* = 136]	*P* value
pH*	7.43 [0.04]	7.43 [0.05]	0.06
PaO_2_*	104 [40]	87 [36]	< 0.001
PaCO_2_*	37 [5]	36 [5]	0.3
SpO_2_*	96 [3]	95 [3]	< 0.001
PaO_2_/FiO_2_*	168 [68]	126 [65]	< 0.001
SaO_2_/FiO_2_*	161 [40]	143 [42]	< 0.001
ROX index*	8.3 [3]	6.8 [2.5]	< 0.001
Heart rate*	82 [15]	82 [14]	0.9
Respiratory rate*	25 [6]	26 [5]	0.07
Flow [Lt/min]*	60 [0.3]	60 [0.4]	0.4
FiO_2_*	0.63 [0.14]	0.72 [0.18]	< 0.001

^*^mean [SD]

### Physiological characteristics after 24 h of APP

After 24 h of APP, patients who required intubation had significantly lower PaO₂ (87 vs. 104 mmHg), PaO₂/FiO₂ ratio (126 vs. 168), SaO₂/FiO₂ ratio (143 vs. 161), SpO₂ (95% vs. 96%), and ROX index (6.8 vs. 8.3) compared to those who did not require intubation (*p* < 0.001 for all). FiO₂ was also higher in the intubated group (0.72 vs. 0.63; *p* < 0.001). No significant differences were observed in pH, PaCO₂, respiratory rate, heart rate, or flow rate (Table 2).

**Table 2:** Physiological characteristics between intubated and non–intubated patients after 24 h of awake proning

Variable	No intubation [*N* = 264]	Intubation [*N* = 136]	*P* value
Age*	53.4 [12.8]	61.5 [10.9]	< 0.001
Gender, male**	194 [73.5]	103 [75.7]	0.72
BMI [kg/m^2^]*	31 [4.9]	30.9 [6]	0.81
Flow [Lt/min]*	60 [0.3]	60 [0.4]	0.5
FiO_2_*	0.61 [0.20]	0.64 [0.18]	0.14
pH*	7.43 [0.04]	7.43 [0.04]	0.72
PaO_2_*	81 [29]	71 [22.4]	< 0.001
PaCO_2_*	36 [5]	35.3 [5]	0.06
SpO_2_*	93 [4.8]	92 [4.4]	0.07
PaO_2_/FiO_2_*	145 [63]	125 [52]	0.004
SaO_2_/FiO_2_*	181 [88]	165 [78]	0.07
ROX index*	7.5 [4]	6.6 [3]	0.03
Heart rate*	82 [15]	82 [14]	0.9
Respiratory rate*	25 [6]	26 [5]	0.07
Total time of the first day of prone position [hours]*	12.5 [4.8]	11.7 [6.90]	0.20
Days in prone position*	5 [4]	3 [2]	< 0.001
Time from admission to the first APP session [hours]*	2 [2]	6 [3.5]	< 0.001
Mortality**			
Day 3	1 [0.4]	2 [1.5]	
Day 7	1 [0.4]	7 [5]	
Day 28	2 [1]	48 [31.8]	

^*^mean [SD], ** n [%]

### Other clinical outcomes

At day 3, 1 out of 264 non–intubated patients (0.4%) and 2 out of 136 intubated patients (1.5%) had died (Table 1). Between days 3 and 7, three additional patients died, followed by 12 more by day 28. Final mortality at day 28 was 48 out of 151 intubated patients (31.8%) and 2 out of 249 non–intubated patients (1.0%) as shown in Table 1.

### Predictors of early intubation

At baseline, three variables were independently associated with intubation within the first 3 days. In the multivariable logistic regression model, older age (OR: 1.05 per year; 95% CI 1.03–1.08; *p* < 0.001), lower PaO₂ (OR: 0.98 per mmHg; 95% CI 0.98–0.99; *p* = 0.03), and higher respiratory rate (OR: 1.08 per breath/min; 95% CI 1.01–1.15; *p* = 0.02) were significantly associated with an increased risk of early intubation.

At 24 h after initiation of APP, a multivariable logistic regression model was developed to predict early intubation (≤ 72 h). The following variables were independently associated with intubation: age (OR: 1.05 per year; 95% CI 1.03–1.08; *p* < 0.001), PaO₂ (OR: 0.98 per mmHg; 95% CI 0.98–0.99; *p* = 0.003), FiO₂ (OR: 10.2; 95% CI 3.1–33.4; *p* < 0.001), SaO₂/FiO₂ (OR: 1.02 per unit; 95% CI 1.00–1.04; *p* = 0.01), respiratory rate (OR: 1.08; 95% CI 1.01–1.15; *p* = 0.02) (Table 3).

**Table 3:** Odds ratios for variables at baseline independently associated with an increased risk of intubation

Variable	Odds ratio [95% CI]	*P* value
Age	1.05 [1.03–1.08]	< 0.001
PaO_2_	0.98 [0.98–0.99]	0.003
FiO_2_	10.2 [101–102]	< 0.001
SaO_2_/FiO_2_	1.02 [1.00–1.04]	0.01
Respiratory rate	1.08 [1.01–1.15]	0.02

AUC [area under the curve] = 0.78 95% CI (0.76–0.81)

### Prediction model and nomogram construction

**Fig. 3:**
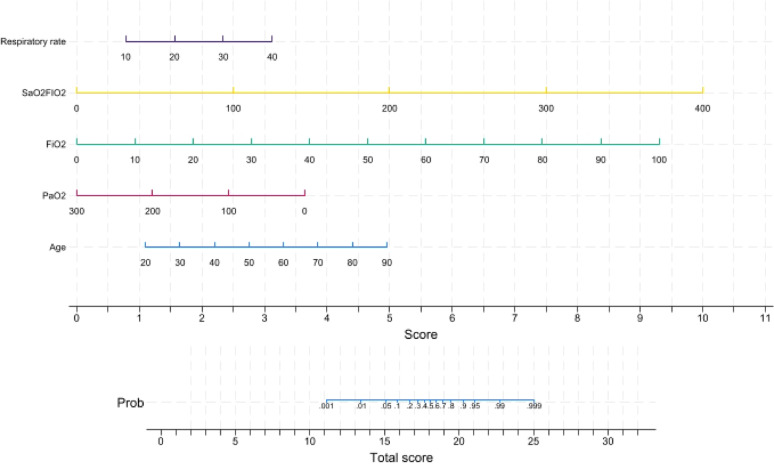
Characteristics in the nomogram to predict the probability of intubation in patients with acute respiratory failure due to COVID–19–ARDS. As an example, a 55–year–old patient is admitted to the ICU under HFO with a flow of 60 L/min and FiO2 of 0.7 in the first 24 h. The patient has a respiratory rate of 25 breaths per minute, SaO2/FiO2 of 128, and a PaO2 of 65 mmHg. The patient’s score was 24,5, and the probability of intubation was more than 95% at day 3

Predictors were derived from physiological measurements obtained 24 h after initiation of awake prone positioning. These variables were combined into a single multivariable model to reflect both initial severity and early response to treatment. Based on this final model, a nomogram was constructed to estimate individual risk of early intubation. Each variable was assigned a weighted score according to its regression coefficient, and the total score was mapped to a predicted probability of intubation (Fig. 3).

A multivariable model evaluating the changes in physiological variables between baseline and 24 h did not provide additional predictive value beyond the models based on absolute values at each time point. As these dynamic changes were not independently associated with early intubation, the model was not retained in the final analysis. In sensitivity analyses, the main model showed robust and consistent discriminative performance (Table S1; Figures S3–S4).

## Discussion

This post hoc analysis of a large cohort of COVID–19 patients with acute respiratory failure who underwent awake prone positioning (APP) within 24 h of ICU admission shows that physiological factors—specifically hypoxemia, elevated respiratory rate, and advanced age—possess moderate predictive value for intubation by day 3. Sensitivity analyses confirmed the robustness of the main model (Table 1S). These findings highlight hypoxemia and increased work of breathing as key clinical markers of disease progression in COVID–19, underscoring the need for close monitoring to identify patients at higher risk of deterioration and to guide timely, individualized interventions aimed at mitigating the need for invasive mechanical ventilation.

While many features of COVID–19–related ARF overlap with other etiologies, the early dissociation between oxygenation and respiratory mechanics, alongside the frequent vascular involvement, render its clinical course sufficiently distinct to merit dedicated risk assessment approaches [22, 23]. In this context, the optimal timing of intubation in patients with COVID–19–related acute respiratory failure—whether earlier or later—has been a subject of ongoing debate. While some observational studies have suggested that later intubation may be associated with adverse outcomes [7, 8, 24], recent methodologically rigorous evidence has challenged this assumption. Emulated target trials in patients with ARF [9], along with indirect evidence from meta–analyses of randomized controlled trials (RCTs) in COVID–19 patients [6], do not support a causal relationship between intubation timing and outcomes such as mortality. These analyses reported no significant difference in mortality between intubated patients in the APP and control arms, despite intubation occurring later in the APP group. However, these findings should be interpreted with caution. As these are post hoc analyses, the subgroup of intubated patients was not randomized, limiting comparability between groups [25]. Furthermore, such comparisons are vulnerable to important methodological limitations, including selection bias, immortal time bias, and unmeasured confounding due to time–dependent clinical trajectories [26, 27]. Given that conducting a randomized controlled trial to test the hypothesis that earlier intubation leads to better outcomes than later intubation would be neither feasible nor ethically appropriate, identifying early those patients unlikely to benefit from prolonged APP and more likely to require intubation appears to be a reasonable and clinically relevant approach to guide timely, individualized decision–making.

The decision to define early intubation (≤ 72 h) as the primary outcome was based on both methodological and clinical considerations. Our objective was not to estimate long–term prognosis, but rather to support early clinical decision–making in patients receiving non–invasive respiratory support. This timeframe is consistent with the principles of diagnostic prediction models, which aim to anticipate imminent deterioration [18]. Furthermore, evidence from the largest randomized trial to date indicates that approximately two–thirds of patients who require intubation do so within the first 72 h [28]. Therefore, this window captures the majority of early failure events, during which risk stratification may help guide individualized and timely interventions.

We acknowledge that the decision to initiate mechanical ventilation—both in general and particularly during the pandemic—is highly variable and influenced by a complex interplay of factors, including ICU admission thresholds, clinicians’ perception of the need for intubation, resource availability, and culturally shaped expectations and preferences for care [9]. This variability must be carefully considered in studies where intubation is used as a clinical endpoint. In our study, we attempted to reduce inter–center heterogeneity by providing detailed, standardized recommendations for intubation and initiation of invasive mechanical ventilation. These recommendations were based on the FLORALI trial [14] and have been described in our previous reports [14–16].

Although some of the predictors included in our model are physiologically related, their simultaneous inclusion is appropriate given the predictive aim of our analysis. Unlike explanatory or prognostic models, predictive models are not designed to estimate the independent effect of each variable, but rather to optimize the model’s ability to anticipate outcomes in new patients. In this context, a certain degree of correlation among variables does not necessarily undermine model performance, particularly when multicollinearity diagnostics are within acceptable limits. Collinearity is generally less concerning in predictive modeling, where accuracy and generalizability are prioritized over inference. Ultimately, our goal is to provide a clinically useful model that can support timely decisions, taking into account both performance metrics and feasibility of implementation. While individual parameters such as respiratory rate or PaO₂ are frequently used in bedside assessment, their integration within a predictive model provides added value by capturing multidimensional aspects of respiratory function that may be missed when assessed in isolation [29].

While our study offers several methodological strengths, it also presents limitations that should be considered. First, this was a prospective, multicenter cohort conducted across six ICUs, enhancing the external validity of our findings and reducing the risk of selection bias. Standardized data collection procedures and centralized oversight ensured high data quality and consistency across sites. The model was built using physiologically justified predictors that are routinely available at the bedside, improving its clinical applicability. Furthermore, the temporal separation between exposure (physiological data at 24 h) and outcome (intubation within 72 h) strengthens the internal validity of the predictive approach. The construction of a nomogram based on this model offers an intuitive tool to estimate individual risk and potentially assist in clinical decision–making. Nonetheless, some limitations should be acknowledged. Although the selected variables were systematically recorded, certain clinically relevant factors—such as signs of respiratory muscle fatigue, frailty, or nuanced clinician judgment—may not have been captured. While their exclusion does not introduce confounding in the traditional etiologic sense, it may limit the model’s predictive accuracy or generalizability in settings not represented in our cohort. This reflects the trade–off between model simplicity, bedside feasibility, and comprehensive variable inclusion. Second, the model underwent internal validation via bootstrap resampling, but external validation in independent cohorts, including non–COVID acute respiratory failure, remains necessary. By excluding patients who required immediate intubation or could not tolerate at least 6 h of awake prone positioning, the study population may underrepresent patients with more severe illness, potentially limiting generalizability to that subgroup. A final limitation of our study is the absence of external validation, as we tested the APP only in COVID–19 patients. However, we believe that our findings may offer a framework for future research to evaluate the potential clinical value of prone positioning in spontaneously breathing patients with ARDS caused by various factors, such as sepsis, bacterial or viral pneumonia, or aspiration of gastric contents.

## Conclusions

The findings of our study show that variables related to oxygenation—particularly those measured 24 h after the initiation of awake prone positioning—are associated with the risk of early intubation in patients with COVID–19–related acute respiratory failure. These parameters reflect the early physiological response to non–invasive support and may provide critical information beyond baseline assessment. Incorporating them into a predictive model may assist clinicians in identifying patients at higher risk of treatment failure and in guiding timely, individualized decisions regarding escalation of care. However, these findings should be externally validated in independent cohorts before the model can be implemented in routine clinical practice.

## Supplementary Information

Below is the link to the electronic supplementary material.

Supplementary Material 1.

## Data Availability

Morales–Quinteros, Esperatti and Fuentes had full access to all data of the study and take responsibility for the integrity and the accuracy of data analysis.

## Abbreviations

APP: Awake prone position
ARDS: Acute respiratory distress syndrome
ARF: Acute respiratory failure
COVID–19: Coronavirus disease 2019
FiO_2_: Fraction of Inspired Oxygen
GCS: Glasgow coma scale
HFNO: High flow nasal oxygen
ICU: Intensive care unit
IMV: Invasive mechanical ventilation
SaO_2_: Saturation of oxygen in arterial blood
SpO_2_: Saturation of oxygen in peripheral blood
PaO_2_: Partial pressure of oxygen in the arterial blood
