# Supplementary material for: A predictive model for early intubation in patients with COVID–19–induced acute hypoxemic respiratory failure under awake prone position
Source: Ann Intensive Care. 2025 Nov 24;15:188. doi: 10.1186/s13613-025-01602-4 (PMC12644287; doi:10.1186/s13613-025-01602-4)
Supplement: Supplementary file 1 — Supplementary Material 1. [file 13613_2025_1602_MOESM1_ESM.docx]

**SUPPLEMENTARY MATERIAL**

[Distribution of variables and model calibration 1](#_Toc207647479)

[FIGURE 1S: Calibration plot of the predictive model 2](#_Toc207647480)

[FIGURE 2S. Cumulative frequency distribution of the study variables 2](#_Toc207647481)

[Performance of alternative predictive models and nomograms 3](#_Toc207647482)

[Table 1S-Performance of alternative predictive models 4](#_Toc207647483)

[Figure 3S- Nomogram SaO2/FiO2 model 4](#_Toc207647484)

[Figure 3S- Nomogram PaO_2_/FiO_2_ model 5](#_Toc207647485)

# **Distribution of variables and model calibration**

## **FIGURE 1S:** Calibration plot of the predictive model

**
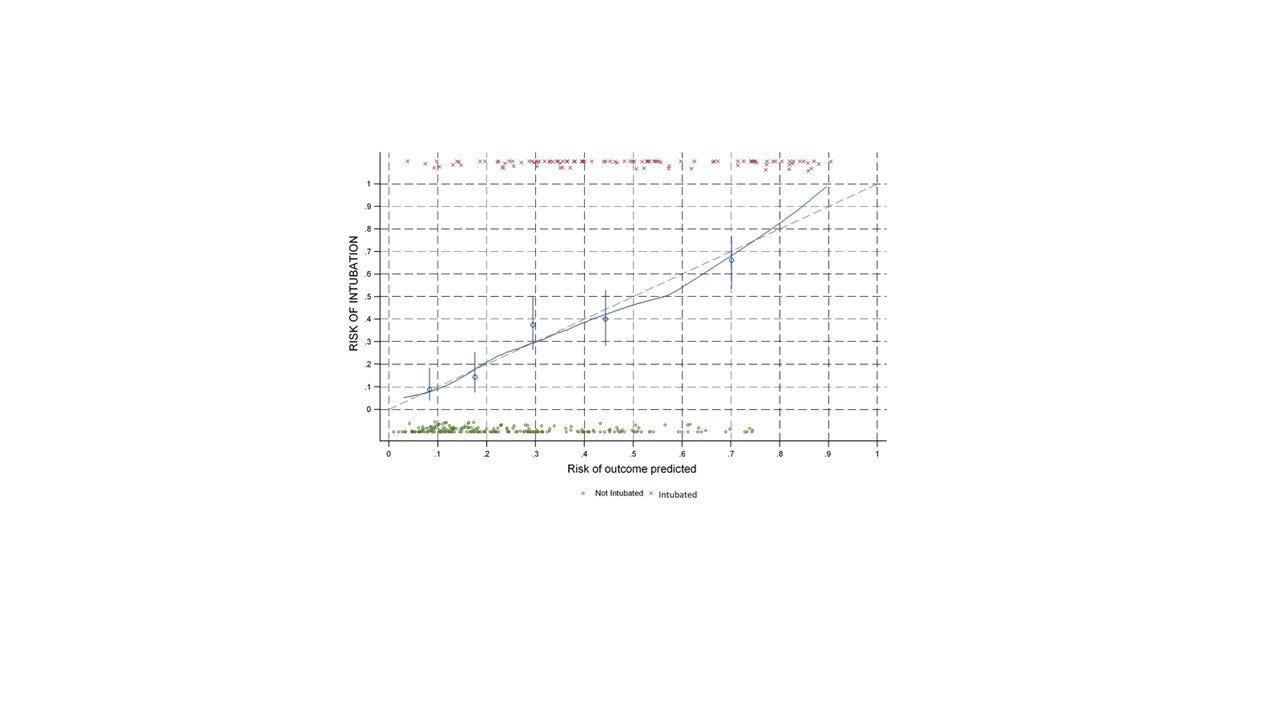
**

## **FIGURE 2S**. Cumulative frequency distribution of the study variables


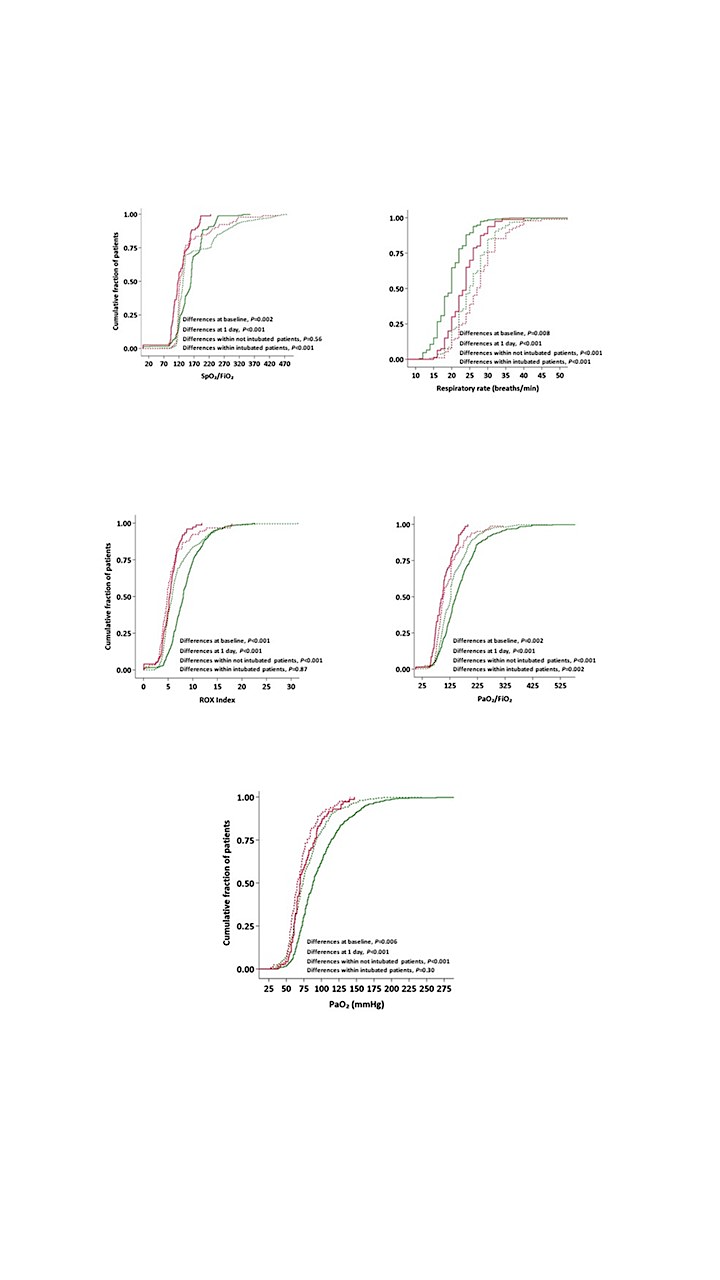


Cumulative frequency distribution of the study variables: SpO2/FiO2, respiratory rate, ROX index, PaO2/FiO2 and PaO2. The plots show the study parameters at baseline and 24 h after awake prone positioning of intubated and non–intubated patients. The red lines represent intubated patients at 24 h of awake prone positioning; the dotted red lines represent intubated patients before awake positioning; green lines represent non–intubated patients at 24 h of awake prone positioning; the dotted green lines represent non–intubated patients before awake prone positioning

# **Performance of alternative predictive models and nomograms**

## **Table 1S-**Performance of alternative predictive models

|  | **AUC** | **95%CI** | **P value** |
| --- | --- | --- | --- |
| **Main model***  (Age, PaO2, FiO2, SaO2/FiO2, Respiratory rate) | 0.78 | 0.73-0.83 | - |
| **SaO2/FiO2 model**  (Age, SaO2/FiO2, Respiratory rate) | 0.73 | 0.71-0.76 | **0.08** |
| **PaO_2_/FiO_2_ model** (Age, PaO2/FiO2, Respiratory rate) | 0.76 | 0.73-0.78 | 0.37 |
| **Δ 24 to baseline** (PaO2, FiO2, SaO2/FiO2, Respiratory rate) | 0.78 | 0.76-0.81 | 1.00 |

*Main model as a reference to compare.

##

## **Figure 3S-** Nomogram SaO₂/FiO₂model

## **Figure 3S**- Nomogram PaO_2_/FiO_2_ model
